# *In Vivo* Potential Anti-Inflammatory Activity of *Melissa officinalis* L. Essential Oil

**DOI:** 10.1155/2013/101759

**Published:** 2013-12-05

**Authors:** Amina Bounihi, Ghizlane Hajjaj, Rachad Alnamer, Yahia Cherrah, Amina Zellou

**Affiliations:** ^1^Laboratory of Pharmacology and Toxicology, Department of Drugs Sciences, Faculty of Medicine and Pharmacy, Mohammed V Souissi University, Rabat Instituts, BP 6203, Agdal, Rabat, Morocco; ^2^Laboratory of Biochemistry and Immunology, Department of Biology, Faculty of Science, Mohammed V Agdal University, Rabat Instituts, BP 6203, Agdal, Rabat, Morocco; ^3^Departement of Pharmacology, University of Thamar, BP 87246, Thamar, Yemen

## Abstract

*Melissa officinalis* L. (Lamiaceae) had been reported in traditional Moroccan medicine to exhibit calming, antispasmodic, and strengthening heart effects. Therefore, this study is aimed at determining the anti-inflammatory activities of *M. officinalis* L. leaves. The effect of the essential oil of the leaves of this plant was investigated for anti-inflammatory properties by using carrageenan and experimental trauma-induced hind paw edema in rats. The essential oil extracted from leaves by hydrodistillation was characterized by means of gas chromatography-mass spectrometry (GC-MS). *M. officinalis* contained Nerol (30.44%), Citral (27.03%), Isopulegol (22.02%), Caryophyllene (2.29%), Caryophyllene oxide (1.24%), and Citronella (1.06%). Anti-inflammatory properties of oral administration of essential oil at the doses of 200, 400 mg/kg p.o., respectively, showed significant reduction and inhibition of edema with 61.76% and 70.58%, respectively, (*P* < 0.001) induced by carrageenan at 6 h when compared with control and standard drug (Indomethacin). On experimental trauma, *M. officinalis* L. essential oil showed pronounced reduction and inhibition of edema induced by carrageenan at 6 h at 200 and 400 mg/kg with 91.66% and 94.44%, respectively (*P* < 0.001). We can conclude that the essential oil of *M. officinalis* L. possesses potential anti-inflammatory activities, supporting the traditional application of this plant in treating various diseases associated with inflammation and pain.

## 1. Introduction

The varied climate and heterogeneous ecologic conditions in Morocco have favoured the proliferation of more than 42,000 species of plants, divided into 150 families and 940 genuses [1–4]. Over the past decade herbal medicine has become a topic of global importance, making an impact on both world health and international trade. Medicinal plants continue to play central roles in the healthcare system of large proportion of the world's population [3]. This is particularly true in the developing countries, where herbal medicine has a long and uninterrupted history of use. Recognition and development of medicinal and economic benefits of these plants are increasing in both developing and industrialized nations. Continuous usage of herbal medicine by a large proportion of the population in the developing countries is largely due to the high cost of western pharmaceuticals, health care, adverse effects that follow their use (in some cases), and the cultural, spiritual point of view of people [5–7]. In western developed countries, however, after a downturn in the pace of herbal use in recent decades, the pace is again quickening as scientists realize that the effective life span of any antibiotic is limited [8–10]. Worldwide spending on finding new anti-infective agents (including vaccines) was expected to increase 60% from the spending levels in 1993. New sources, especially plant sources, are also being investigated. Secondly, the public is becoming increasingly aware of problems with the overprescription and misuse of traditional antibiotics. In addition, many people are interested in having more autonomy over their medical care. All these make the knowledge of chemical, biological, and therapeutic activities of medicinal plants used as folklore medicine become necessary [11–14].

Generally, the inflammatory process involves a series of events that can be elicited by numerous stimuli such as infectious agents, ischemia, antigen-antibody interaction, and thermal or physical injury. Inflammation is usually associated with pain as a secondary process resulting from the release of analgesic mediators: nonsteroidal anti-inflammatory drugs (NSAIDs), steroidal drugs, and immunosuppressant drugs, which have been used usually in the relief of inflammatory diseases by people around the world for a long time [9].

However, these drugs were often associated with severe adverse side effects, such as gastrointestinal bleeding and peptic ulcers [9]. Recently, many natural medicines derived from medicinal plants were considered as effective and safer for the treatment of various diseases including inflammation and pain [15].

There are various components to an inflammatory reaction that can contribute to the associated symptoms and tissue injury. Edema formation, leukocyte infiltration, and granuloma formation represent such components of inflammation [16]. Edema formation in the paw is the result of a synergism between various inflammatory mediators that increase vascular permeability and/or the mediators that increase blood flow [17, 18].

*M. officinalis* L. (Lamiaceae) is a herbal medicine native to the Eastern Mediterranean region and Western Asia. *M. officinalis* has been traditionally used for different medical purposes such as tonic, antispasmodic medicine drug, carminative, diaphoretic medicine drug, surgical dressing for wounds, and sedative/hypnotic, and it is used for strengthening the memory and relief of stress-induced headache [19–21]. In our previous study, we have proven the efficacy of the extract of the essential oil of this plant on central nervous activity [22]. To the best of our knowledge, this is the first study to provide data that the essential oil of the leaves of *M. officinalis* L. evaluated against inflammations. Thus, the aim of this study is to evaluate the anti-inflammatory effect of the essential oil of the leaves of *M. officinalis* L. and, therefore, to determine the scientific basis for its use in traditional medicine in the treatment of inflammation.

## 2. Materials and Methods

### 2.1. Plant Material

Fresh leaves of *Melissa officinalis* L. (Lamiaceae) were collected based on ethnopharmacological information from villages around the region Eljadida, middle Morocco in January 2013, with the agreement from the authorities with respect to the United Nations Convention of Biodiversity and with assistance of traditional medical practitioner. The plant was identified with botanist of the Department of Medicinal and Aromatic Plants, National Institute for Agricultural Research, Morocco. A voucher specimen (no. RAB76712) was deposited in the Herbarium of Botany Department of Scientific Institute of Rabat.

### 2.2. Preparation of the Essential Oil

Fresh leaves of *Melissa officinalis* L. were hydrodistilled in Clevenger apparatus for 4 hours to obtain the essential oil with (v/w) yield. The extract was stored in a refrigerator at 4°C [23] and protected against light and heat until use. The essential oil was produced from leaves of *M. officinalis* by hydrodistillation method. Plant materials (100 g) cut into small pieces were placed in distillation apparatus and hydrodistilled for 4 h after the oils were dried over hydrous K_2_CO3; they were stored at +4°C until used for GC-MS analysis. The yield of extraction (ratio weight of EO/weight of dry plant) was 0.5% [1–3].

### 2.3. Phytochemical Analysis of *Melissa officinalis* L. Essential Oil by Combined Gas Chromatography-Mass Spectrometry (GC-MS)

The essential oil was submitted to quantitative analysis in a Hewlett-Packard 575, GC condition: carrier gas N2 (0.5 bar) at flow rate of 1.0 m/min, sample size: 0.2 *μ*L injected, and capillary column (30 m siloxane 5% HP EM). The temperature of the injector and detector was set at 250°C. The oven temperature was programmed from 50°C to 250°C (5 min). The MS was taken at 70 eV. The components of the essential oil were identified by comparison of their mass spectra with those in the Wiley-NIST 7th edition library of mass spectral data. The percentage composition on the oil sample was calculated from GC-MS peak areas [1, 2].

### 2.4. Animals

Male Wistar rats weighing 180–220 g were used in this study. The animals were obtained from the animal centre of Mohammed V-Souissi University, Medicine and Pharmacy Faculty, Rabat, Morocco. All animals were kept in a room maintained under environmentally controlled conditions of 23 ± 1°C and 12 h light-12 h dark cycle. All animals had free access to water and standard diet. They were acclimatized at least one week before the experiments started. The animals submitted to oral administration of the extracts or drugs were fasted for 18 h before the experiment (water was available). All experiments were conducted in accordance with the Official Journal of the European Committee in 1991. The experiment protocol was approved by the Institutional Research Committee regarding the care and use of animals for experimental procedure in 2010; CEE509 [1–3, 24, 25].

### 2.5. *In Vivo* Anti-Inflammatory Activity

The evaluation of the anti-inflammatory activity of *M. officinalis* L. essential oil was carried out by using two different methods that used chemical stimuli (winter test) [26] and mechanical stimuli (Riesterer and Jacques test) [27] induced paw edema in rats. In both methods, all animals were fasted 18 h before testing and received 5 mL of distilled water by gavages to minimize individual variations in response to the swelling of the paws. The right hind paw (RP) is not treated, and it is taken as control.

### 2.6. Carrageenan-Induced Rat Paw Edema

The carrageenan-induced paw edema model [9, 26–28] was used to evaluate the anti-inflammatory effect of *M. officinalis *essential oil. The initial paw volume was recorded using an Ugo Basile model LE750 plethysmometer.

Rats groups were orally administered essential oil of *M. officinalis *L. (200 and 400 mg/kg); Indomethacin (10 mg/kg) was used as reference drug while distilled water (5 mL/kg) was used as negative control. After 60 min had passed, carrageenan (0.05 mL of a 1% w/v solution, prepared in sterile saline) was injected subcutaneously into subplantar region of the left hind paw of each rat. The right hind paw is not treated; it is taken as a witness. One hour 30 minutes, 3 hour and 6 hours after the injection of carrageenan, the paw volumes of each rat were measured. Mean differences of treated groups were compared with the mean differences of the control group. The percentages of inhibition of inflammation were calculated according to the following formula:

(1) %  of  inhibition =mean  [Vleft−Vright]control−[Vleft−  Vright]treated[Vleft−Vright]control  ∗100,

where *V*_left_ is the mean volume of edema on the left hind paw and *V*_right_ is the mean volume of edema on the right hind paw.

### 2.7. Experimental Trauma-Induced Rat Paw Edema

This assay was determined as described by Riesterer and Jacques test [27]. The test groups of rats were given orally 200 and 400 mg/kg of *M. officinalis *essential oil, the control group received 5 mL/kg of distilled water, and the standard group received the reference drug (Indomethacin 10 mg/kg). One hour after oral administration of different substances dropping a weight of 50 g onto the dorsum of the left hind paw of all animals. The right hind paw is not treated; it is taken as a witness.

The difference volume of two paws was measured and taken as the edema value by using digital plethysmometer LE750 at 1 h 30 min, 3 h and 6 h after induction of inflammation [29]. Mean differences of treated groups were compared with the mean differences of the control group. The percentages of inhibition of inflammation were calculated according to the following formula:

(2) %  of  inhibition =mean  [Vleft−Vright]control−[Vleft−  Vright]treated[Vleft−Vright]control  ∗100.

### 2.8. Statistical Analysis

The results are expressed as mean ± SEM and analyzed by one-way analysis of variance (ANOVA) followed by Student's *t*-test. A value of *P* < 0.001 was considered significant.

## 3. Results

### 3.1. Chemical Composition of the Essential Oil

**Figure 1:**
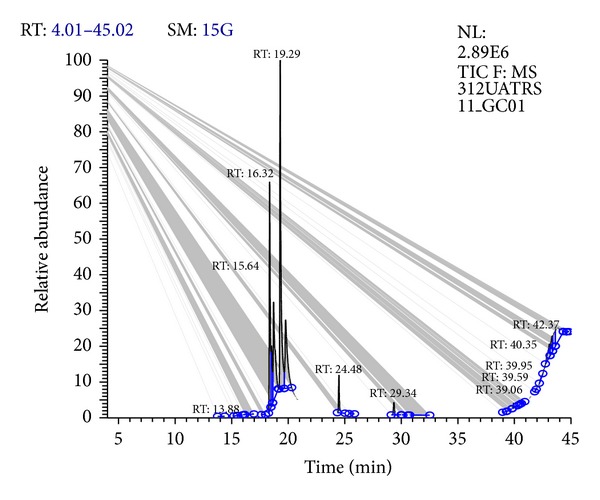
Gas chromatography-mass spectrometry (GC-MS) of *Melissa officinalis *L. essential oil.

The results obtained by GC-MS analyses of the essential oils of *Melissa officinalis *L. are presented in Figure 1. Thirteen compounds were identified in this essential oil by GC-MS analyses (Figure 1); *M. officinalis *L. contained six major compounds, that is, Nerol (30.44%), Isopulegol (22.02%), Citral (27.03%), Caryophyllene (2.29%), Caryophyllene oxide (1.24%), and Citronella (1.06%) as the main constituents of essential oil of *M. officinalis* L. Nerol and Citral as have been previously reported major chemical components of *M. officinalis *L. [18–22], but Isopulegol has never been reported as the main component of *M. officinalis *L. (Figure 1).

### 3.2. Carrageenan-Induced Rat Paw Edema

The results of the effect of the *M. officinalis *essential oil on carrageenan-induced edema are shown in Tables 1 and 2. At doses of 200 and 400 mg/kg via oral pathway, *M. officinalis *essential oil exhibited significant (*P* < 0.001) anti-inflammatory activity as compared to the control and standard group (Table 1). At 1 h 30 min, the extract of the essential oil showed similar inhibition of edema by 70.58% and 76.47% at 200 and 400 mg/kg, respectively, as compared to the standard drug Indomethacin (10 mg/kg) by 76.47%. However, at the sixth hour the *M. officinalis *essential oil showed greater inhibition with 61.76% and 70.58% at 200 and 400 mg/kg, p.o., respectively, as compared to reference drug Indomethacin (10 mg/kg) by 52.94% (Table 2).

### 3.3. Experimental Trauma-Induced Rat Paw Edema

The effect of two doses of the *M. officinalis *essential oil on experimental trauma-induced inflammation is shown in Tables 3 and 4, and the results are comparable to that of the control and standard drug Indomethacin (10 mg/kg, p.o.). The *M. officinalis *essential oil at all DOE levels significantly decreased inflammation induced by experimental trauma (Table 3).

At 200 and 400 mg/kg, p.o., *M. officinalis *essential oil exhibited maximum anti-inflammatory activity of 91.66% and 94.44%, respectively, at the sixth hour (Table 4). This inhibition of edema was significantly similar to that obtained with Indomethacin (10 mg/kg, p.o.) by 91.66% during the same time.

## 4. Discussion

Aromatic and medicinal plants have been used for thousands of years in every part of the world by numerous civilizations. Driven by their intuition and their sense of observation, they were able to find answers to their health problems in the plant environment [4–6]. Recently, the search for novel pharmacotherapy from medicinal plants for inflammation diseases has progressed significantly owing to their less side effects and better tolerability. Aromatherapy is currently used worldwide in the management of chronic pain [1–5]. Oil compositions of *M. officinalis* L. have already been reported [18–22]. Thus, it has been shown that Nerol, Isopulegol, Citral, Caryophyllene, Caryophyllene oxide, and Citronella account for 80% of *M. officinalis* L. essential oils, but in our study, these compounds represent 84.08%. These differences in chemical composition of essential oil may be due to both developmental and environmental factors that influence plant metabolism. We analyzed the effects of different doses of essential oil from leaves of *M. officinalis* L. for their anti-inflammatory activity.

Following oral administration of *M. officinalis* L. extract at the doses of 300 and 2000 mg/kg, p.o., no toxicity and no significant changes in the body weight between the control and treated groups were demonstrated at these doses. This result indicates that the LD_50_ was higher than 2000 mg/kg. These results were previously reported by Bounihi et al. [22].

In the present study, anti-inflammatory effect of the essential oil of *M. officinalis* L. was investigated after subplantar injection of carrageenan and experimental trauma in rat paw.

The method of carrageenan induced paw edema is the most widely used to evaluate the anti-inflammatory effect of natural products. Edema formation due to carrageenan in the rat paw is the biphasic event [30]. The initial phase, which occurs between 0 and 2.5 h after the injection of the phlogistic agent, has been attributed to the action of mediators such as histamine, serotonin and bradykinin on vascular permeability [30–32]. It has been reported that histamine and serotonin are mainly released during the first 1.5 h while bradykinin is released within 2.5 h after carrageenan injection [32, 33]. While the late phase is associated with the release of prostaglandins and may be occurs from 2.5 h to 6 h post-carrageenan injection [33, 34].

In the inflammatory response there is an increase of permeability of endothelial lining cells and influxes of blood leukocytes into the interstitium, oxidative burst, and release of cytokines (interleukins and tumors necrosis factor-*α* (TNF-*α*)). At the same time, there is also an induction of the activity of several enzymes (oxygenases, nitric oxide synthases, and peroxidases) as well as the arachidonic acid metabolism. In the inflammatory process there is also the expression of cellular adhesion molecules, such as intercellular adhesion molecule (ICAM) and vascular cell adhesion molecule (VCAM) [34].

The carrageenan-induced hind paw edema in rat is known to be sensitive to cyclooxygenase inhibitors, but not to lipooxygenase inhibitors, and has been used to evaluate the effect of nonsteroidal anti-inflammatory drugs which primarily inhibit the cyclooxygenase involved in prostaglandins synthesis. It has been demonstrated that the suppression of carrageenan-induced inflammation after the third hour correlates reasonably with therapeutic doses of most clinically effective anti-inflammatory agents [33].

*M. officinalis *essential oil at doses of 200 and 400 mg/kg, p.o., reduced and inhibited significantly (*P* < 0.001) the edema in the early and late phases of inflammation induced by carrageenan. In the experimental trauma-induced edema in rats, the extract was also reduced and inhibited significantly (*P* < 0.001) the edema in the different phases of inflammatory response. Based on the results obtained, *M. officinalis *essential oil was able to effectively inhibit the increase in paw volume during the phases of inflammation. This indicates that the extract of the *M. officinalis *essential oil, has a significant anti-inflammatory activity perhaps by inhibiting the release of the inflammatory mediators; serotonin and histamine also suppressed prostaglandin and cytokine.

Carrageenan and experimental trauma-induced paw edema in rats are a suitable experimental animal model to evaluate the antiedematous effect of diverse bioactive compounds such as plant extracts and essential oils [35–38]. If this method allows screening the anti-inflammatory of samples, very little information is given about its mechanism.

The exact mechanism of the anti-inflammatory activity of the essential oil used in the present study is unclear. However, other investigators have reported that the* M. officinalis *essential oil contains Nerol, Citral, Caryophyllene and Citronella as the main components. The same phytochemicals also investigated in this extract by us [22]. Citral constitutes the main components of *Cymbopogon citrates* stapf essential oil. This EO is revealed to be capable to suppress IL-1*β* and IL-6 in LPS-stimulated peritoneal macrophages of normal mice [37, 38]. Whether some essential oils are able to inhibit the production of proinflammatory cytokines such as TNF-*α*, some of them, their main components (Citral, geraniol, citronellol, and carvone), can also suppress TNF-*α*-induced neutrophil adherence responses [38]. Another work [39] revealed that Citral inhibited TNF-*α* in RAW 264.7 cells stimulated by lipopolysaccharide. According to these authors, the *M. officinalis *essential oil we used can be associated with anti-inflammatory activity at least due to the presence of the Citral as the main component.

Further chemical and pharmacological analysis of the extract will be conducted to isolate and characterize the active principles responsible for the anti-inflammatory activity. We can conclude that the essential oil of *M. officinalis* L. possesses potential anti-inflammatory activities, supporting the traditional application of this plant in treating various diseases associated with inflammation and pain.

## Figures and Tables

**Table 1:** Effect of essential oil of *Melissa  officinalis* L. on carrageenan-induced rat paw edema.

Treatment groups	Dose mg/kg p.o.	Mean volume of edema (left paw-right paw) induced by carrageenan (mL)		
		1 h 30 min	3 h	6 h
Control		0.17 ± 0.013	0.26 ± 0.01	0.34 ± 0.023
Indomethacin	10	0.04 ± 0.004*	0.07 ± 0.005*	0.16 ± 0.008*
EOMO	200	0.05 ± 0.009*	0.1 ± 0.011*	0.13 ± 0.01*
EOMO	400	0.04 ± 0.01*	0.09 ± 0.008*	0.1 ± 0.013*

Values are expressed as mean ± S.E.M. (*n* = 6), p.o.: oral route, *n*: number of animals per group, EOMO: essential oil of *Melissa  officinalis  *L., *P < 0.001 statistically significant relative to the control and reference drug (Indomethacin).

**Table 2:** Percentage of inhibition of inflammation of essential oil of *Melissa  officinalis* L. using carrageenan-induced rat paw edema.

Treatment groups	Dose mg/kg p.o.	Percentage of inhibition of inflammation induced by carrageenan (%)		
		1 h 30 min	3 h	6 h
Indomethacin	10	76.47	73.07	52.94
EOMO	200	70.58	61.53	61.76
EOMO	400	76.74	65.38	70.58

*N* = 6; these results compared with standard drug (Indomethacin, 10 mg/kg, p.o.) were administered by the oral route.

**Table 3:** Effect of essential oil of *Melissa  officinalis  *L. on experimental trauma-induced rat paw edema.

Treatment groups	Dose mg/kg p.o.	Mean volume of edema (left paw-right paw) induced by experimental trauma (mL)		
		1 h 30 min	3 h	6 h
Control		0.153 ± 0.005	0.22 ± 0.021	0.36 ± 0.015
Indomethacin	10	0.04 ± 0.009*	0.03 ± 0.01*	0.03 ± 0.01*
EOMO	200	0.085 ± 0.005*	0.05 ± 0.007*	0.03 ± 0.005*
EOMO	400	0.068 ± 0.004*	0.04 ± 0.004*	0.02 ± 0.005*

Values are expressed as mean ± S.E.M. (*n* = 6), EOMO: essential oil of *Melissa  officinalis  *L., *P < 0.001 statistically significant compared to the control and reference drug (Indomethacin).

**Table 4:** Percentage of inhibition of inflammation of essential oil of *Melissa  officinalis* L. using experimental trauma-induced rat paw edema.

Treatment groups	Dose mg/kg p.o.	Percentage of inhibition of inflammation induced by experimental trauma (%)		
		1 h 30 min	3 h	6 h
Indomethacin	10	65.81	86.36	91.66
EOMO	200	44.44	77.27	91.66
EOMO	400	55.55	81.81	94.44

*N* = 6; these results compared with standard drug (Indomethacin, 10 mg/kg, p.o.) were administered by the oral route.
